# Identifying neural oscillation and phase synchronization abnormalities in migraine and their predictive values in transcranial magnetic stimulation

**DOI:** 10.22514/jofph.2026.041

**Published:** 2026-05-12

**Authors:** Yujun Wang, Yazhen Han, Manli Huang, Zhongming Gao, Lingxiao Wang, Xiaolian Ma, Qing Zhang, Xianwei Che, Bolin Tan, Jiqing He

**Affiliations:** ^1^Centre for Cognition and Brain Disorders, Hangzhou Normal University Affiliated Hospital, 311121 Hangzhou, Zhejiang, China; ^2^Department of Psychiatry, First Affiliated Hospital, Zhejiang University School of Medicine, 310058 Hangzhou, Zhejiang, China; ^3^Department of Neurology, Hangzhou Normal University Affiliated Hospital, 310000 Hangzhou, Zhejiang, China; ^4^Department of Obstetrics, Hangzhou Normal University Affiliated Hospital, 310000 Hangzhou, Zhejiang, China; ^5^School of Clinical Medicine, Hangzhou Normal University, 311121 Hangzhou, Zhejiang, China

**Keywords:** Migraine, TMS, EEG, PAF, Alpha band

## Abstract

**Background**: Migraine is a prevalent neurological disorder that may substantially disrupt daily function. Repetitive transcranial magnetic stimulation (rTMS) has been shown to be a promising treatment for migraine, but its treatment efficacy still needs to be optimised. This study aimed to identify abnormal neural oscillations in patients with migraines and evaluate their predictive value for TMS treatments to obtain insights for the optimisation of rTMS paradigms. **Methods**: Patients with migraine received a course of rTMS delivered over the left dorsolateral prefrontal cortex (DLPFC). Resting-state electroencephalography (EEG) was assessed at baseline in both patients with migraine and age- and sex-matched healthy controls. **Results**: Compared with healthy controls, patients with migraine were characterised by a slower peak alpha frequency (PAF). In addition, patients with migraine demonstrated alpha-band hyperconnectivity between parieto-occipital regions and between fronto-occipital areas. More importantly, parieto-occipital connectivity showed predictive value for rTMS analgesic effects in this population. **Conclusions**: These findings support the potential utility of oscillatory biomarkers for optimising rTMS treatment in patients with migraine. **Clinical Trial Registration**: Chinese Clinical Trials Registry (ChiCTR2200060337).

## 1. Introduction

Migraine is a prevalent neurological disorder causing significant disability worldwide [1, 2]. During migraine attacks, patients may experience severe, throbbing headaches accompanied by nausea and sensitivity to light and sound, all of which can markedly disrupt daily activities [3]. Although pharmacologic therapy remains central to migraine management, additional treatment options are being developed to reduce migraine frequency and intensity, among which repetitive transcranial magnetic stimulation (rTMS) has attracted increasing attention as a safe and non-invasive brain stimulation approach for migraine. For example, several studies have reported beneficial effects of rTMS in migraine by targeting the dorsolateral prefrontal cortex (DLPFC) [4, 5], and this region has also been linked to analgesic effects across multiple pain modalities in rTMS studies [5, 6, 7, 8].

10-Hz stimulation and intermittent theta burst stimulation (iTBS) are two commonly used forms of rTMS. Among these approaches, 10-Hz rTMS has been widely used for pain management, including in migraine. iTBS is an established rTMS protocol that can modulate neural excitability and increase cortical excitability through a burst stimulation mode [9]. In migraine, only one iTBS study has been reported to date, and it showed a significant reduction in headache frequency and intensity compared with sham stimulation [10]. Moreover, our recent study indicated that 10-Hz rTMS and iTBS may share an analgesic mechanism involving increased gamma oscillations [11]. Consistent with this, our recent meta-analysis of rTMS, which included both 10-Hz and iTBS protocols, demonstrated a generally consistent mid-term therapeutic benefit in migraine, typically defined in systematic reviews of pain studies as 1–6 weeks post-treatment [12]. Nevertheless, treatment response varied considerably across patients and across studies, and the long-term efficacy of rTMS beyond 6 weeks post-treatment remains uncertain in patients with migraine [12]. Collectively, these findings underscore the need to optimise rTMS protocols to enhance clinical outcomes in migraine.

Identifying neural correlates of migraine and rTMS response represents an important strategy to optimise rTMS treatment for migraineurs. In this regard, electroencephalography (EEG) represents a promising technology and is widely available. Among EEG measures, resting-state peak alpha frequency (PAF) has been proposed as a potential biomarker related to pain experiences [13, 14, 15]. Numerous studies have identified a slower PAF in both provoked pain and chronic pain conditions [16, 17, 18]. In migraineurs, decreased PAF was further found to be associated with longer disease duration and longer headache attack duration [19]. These findings suggest that PAF may serve as a biomarker to optimise rTMS efficacy for migraineurs. Although a recent study reported that a 5-day course of rTMS increased PAF, this change did not produce a direct reduction in experimentally induced prolonged pain [20]. Moreover, in that study, PAF was assessed only at pre- and post-treatment, leaving the lasting effect on PAF and its role in analgesia unclear. Therefore, further research is required to clarify the role of PAF in informing personalized rTMS treatment for migraineurs.

In addition to PAF, other neural oscillations have also been investigated in relation to pain experiences. A wide range of abnormal resting-state oscillations have been identified in migraineurs, including delta (1–4 Hz), theta (4–8 Hz), and alpha (8–13 Hz) bands [19, 21, 22]. However, in a large study involving nearly 800 migraineurs, excessive occipital alpha power was identified as a reliable biomarker for migraine [23]. Moreover, occipital alpha power was found to predict treatment response to flunarizine (a calcium channel blocker) in migraineurs, such that responders (≥50% reduction in monthly headache days) had lower baseline alpha power than non-responders [21]. In addition, a recent meta-analysis reported abnormal alpha-band connectivity in migraineurs compared with healthy controls, with 3 of 5 included studies indicating lower alpha-band connectivity in migraineurs [22]. Collectively, these findings highlight the potential utility of alpha power and synchronisation in the diagnosis of migraine and in guiding personalised treatment strategies.

The current study used resting-state EEG to characterise treatment responses to rTMS in migraineurs. We focused on the predictive value of alpha oscillations, given their established role in migraine. A group of migraineurs received rTMS treatment over the left DLPFC. Multidimensional pain experience was assessed, including pain, affective symptoms, and sleep quality, which generally interact with each other in migraine conditions [24]. Resting-state EEG was also evaluated at baseline in both migraineurs and a group of age- and gender-matched healthy controls. It was hypothesised that migraineurs would demonstrate a slower PAF and excessive alpha power compared with healthy controls, and that these abnormal features would be associated with rTMS treatment effects on pain experiences.

## 2. Materials and methods

### 2.1 Study design

This study represents a secondary analysis of a clinical trial. Data collection was conducted from May 2022 to December 2023. Resting-state EEG data were used in the present analysis to examine their relationships with treatment effects on pain experiences. Migraineurs were assigned to either the conventional 10-Hz rTMS group or the iTBS group. Over a two-week period, participants in each group received a course of rTMS treatment (10-Hz or iTBS), with sessions scheduled at least 24 hours apart. Clinical assessments were performed before the intervention (“pre-treatment”), 1 month after the first session (“post-treatment”), and 2 months after the first session (“follow-up”). Resting-state EEG recordings were obtained at pre-treatment.

### 2.2 Participants

Migraine patients were recruited from the Affiliated Hospital of Hangzhou Normal University. The inclusion criteria were: (i) an International Classification of Diseases (ICD-11) diagnosis of migraine [25]; (ii) age ≥15 years; (iii) no changes in medication during the trial or within 2 weeks prior to enrolment; (iv) ability to complete the clinical assessments and rTMS treatment; and (v) willingness to participate and to provide written informed consent. The lower age limit was set at 15 years because migraine can occur in adolescents [26, 27]; however, no participants younger than 18 years were recruited.

The exclusion criteria were: (i) relative or absolute contraindications to TMS intervention [28], including a cardiac pacemaker, cranial metal implant, history of seizure, or current pregnancy; (ii) severe mental disorders (Hamilton Depression Rating Scale (HAMD) score ≥35 or Hamilton Anxiety Rating Scale (HAMA) score ≥29); (iii) aphasia or cognitive impairment (Mini-Mental State Examination score ≤23); (iv) a history of brain surgery, regardless of the indication; and (v) severe cardiopulmonary dysfunction, extreme weakness, or other unstable clinical conditions.

Sample size calculation for the original trial was based on the primary outcome of headache frequency from pre- to post-treatment. For the present analysis, a power analysis was conducted with PAF as the outcome measure using G*Power (3.1.9.6, Heinrich-Heine-Universität Düsseldorf, Düsseldorf, NRW, Germany) [29]. The expected group difference in PAF between chronic pain and healthy controls was used to estimate the required sample size. The analysis indicated that 23 participants per group would be required (α = 0.05, power = 0.95, mean_group1 = 8.6, mean_group2 = 9.4) [30]. Therefore, the sample size in the present study (29 *vs.* 29) was sufficient to achieve adequate statistical power.

A total of 60 patients were screened, of whom 12 did not meet the inclusion criteria, and 6 declined participation. Consequently, 42 participants were assigned to 10-Hz rTMS (n = 23) or iTBS (n = 19). Among them, 30 patients agreed to provide EEG data; however, the EEG data from one participant were damaged. Therefore, EEG data from 29 patients were included in the final analysis (mean age: 38.07 ± 13.73 years; 25 females and 4 males). In addition, 29 healthy controls (mean age: 41.03 ± 12.37 years; 25 females and 4 males) were recruited to match the migraineurs by age and sex (Fig. 1; Table 1). It should be noted that HAMD and HAMA were used only for screening and were not included as continuous covariates, whereas 9-item Patient Health Questionnaire (PHQ-9) and 7-item Generalized Anxiety Disorder scale (GAD-7) were used to evaluate treatment effects on depression and anxiety (Table 1).

**Fig. 1.** Flow diagram of the participants. CONSORT: Consolidated Standards of Reporting Trials; rTMS: Repetitive transcranial magnetic stimulation; iTBS: intermittent theta burst stimulation; EEG: electroencephalography.

**Table 1.** Demographic and clinical characteristics of the participants.

Variables		Migraineurs (n = 29)	Controls (n = 29)	*p *value
Demographics				
	Age (yr, M ± SD)	38.07 ± 13.73	41.03 ± 12.37	0.391
	Gender (Male:Female)	4:25	4:25	1.000
Clinical features				
	Pain types (Episodic:Chronic)	14:15		
	Disease duration (mon, M ± SD)	135.40 ± 141.97		
	Migraine subtype (without:with Aura)	13:16		
	Use of preventive medications (Y:N)	20:9		
	Headache-free before EEG (d)	0–14		
	Monthly headache days (M ± SD)	16.10 ± 9.61		
	Headache intensity (VAS, M ± SD)	6.59 ± 1.84		
	SF-MPQ	16.32 ± 6.13		
	GAD-7	6.99 ± 5.57		
	PHQ-9	8.35 ± 6.52		
	PSQI	9.90 ± 4.78		

*EEG: electroencephalography; M: Mean; SD: Standard Deviation; Y: Yes; N: No; SF-MPQ: short-form McGill Pain Questionnaire; GAD-7: 7-item Generalized Anxiety Disorder scale; PHQ-9: 9-item Patient Health Questionnaire; PSQI: Pittsburgh Sleep Quality Index; VAS: visual analogue scale.*

### 2.3 Clinical assessments

Clinical outcome measures were reported following the Initiative on Methods, Measurement, and Pain Assessment in Clinical Trials (IMMPACT) recommendations for chronic pain clinical trials [31]. The primary outcome measures were headache days and headache intensity in the past month [32]. Secondary outcome measures were employed to capture the multidimensional nature of the patient experience, encompassing pain, affective symptoms, and sleep quality. The short-form McGill Pain Questionnaire (SF-MPQ) was used to evaluate the sensory and affective dimensions of pain, and it demonstrates high test-retest reliability and strong construct validity [33, 34]. The 7-item Generalized Anxiety Disorder scale (GAD-7) was used to measure anxiety symptom severity, and it exhibits excellent internal consistency and strong criterion validity against structured clinical interviews [35, 36]. The 9-item Patient Health Questionnaire (PHQ-9) was utilised to assess depressive symptom severity, and it has high internal consistency and excellent criterion validity, aligning closely with diagnoses made by mental health professionals [37, 38]. The Pittsburgh Sleep Quality Index (PSQI) was administered to measure sleep quality and disturbances, and it has good internal consistency and test–retest reliability in clinical populations [39, 40].

### 2.4 Acquisition of EEG data

EEG recordings were taken in a temperature-controlled, sound-attenuated, and electrically shielded room. Participants sat in a chair with their eyes open and looking forward, and it is noted that migraineurs did not experience headaches during the EEG recording. A 64-channel EEG cap (Brain Products GmbH, Germany) was used to record continuous EEG at 5000 Hz, with FCz (Fronto-Central, zero) and AFz (Antero-Frontal, zero) as the reference and ground electrodes, respectively. EEG impedances were kept below 5 kΩ throughout the recordings. EEG recordings took place in the afternoon for consistency. EEG data were recorded for 5 minutes in accordance with the PAF literature on pain experiences [13, 14, 16, 17, 19, 20, 41].

### 2.5 rTMS treatment

Each session started with obtaining the resting motor threshold (RMT), defined as the minimum stimulation intensity required to induce motor-evoked potentials (MEPs) >0.05 mV in 5 of 10 trials. Single pulses were delivered to the hand region of the left Primary Motor Cortex (M1, 45° to the midline, with the handle pointing backward) using a figure-eight coil connected to a Magstim Rapid2 system (Magstim Company Ltd, Whitland, UK). MEPs were recorded from the first dorsal interosseous (FDI) muscle of the right hand.

rTMS was delivered to the left DLPFC at an intensity of 100% RMT [42]. The left DLPFC was located using the Beam F3 methodology [43], which optimises coil repositioning between sessions [44, 45]. The 10-Hz rTMS protocol consisted of one session per day on weekdays for two weeks (10 sessions in total), and each session included 36 trains of 5-second duration with 25-second inter-train intervals (1800 pulses delivered over 18 minutes). The iTBS paradigm consisted of six sessions per day (with 50-minute inter-session intervals) administered on weekdays for 5 days. Each iTBS session included 10 bursts of 3 pulses delivered at 50 Hz, with a 2-second stimulation period and an 8-second inter-burst interval (600 pulses delivered over 3 minutes) [9]. Thus, both treatments delivered a total of 18,000 pulses.

### 2.6 EEG preprocessing and analysis

EEG data were pre-processed offline using custom-written scripts that implemented functions from EEGLAB (version 13.6.5b, Swartz Center for Computational Neuroscience, La Jolla, CA, USA) [46], running under Matlab R2017b (The MathWorks, Inc., Natick, MA, USA). Data from malfunctioning channels were visually inspected and removed. Butterworth filters (band-pass: 0.5–100 Hz; band-stop notch filter: 48–52 Hz) were then applied to the data [47]. Continuous data were segmented into 4-second non-overlapping epochs. The segmented data were re-referenced to the average reference, and the fast independent component analysis algorithm (FastICA) was applied to remove stereotyped artefacts, including eye blinks, lateral eye movements, muscle activity, and line noise [48]. Missing channels were then interpolated, and epochs were inspected again to remove any anomalous activity in the signal.

EEG frequency representations were calculated using the multitaper method fast Fourier transform (“mtmfft”), as implemented in the FieldTrip toolbox (Donders Institute for Brain, Cognition and Behaviour, Nijmegen, the Netherlands), in the range of 0.5–100 Hz [49]. PAF was defined as the frequency with the highest power within the alpha range (8–13 Hz) [50]. left occipital cortex (O1) and right occipital cortex (O2) were specified as the channels for PAF calculation based on the literature [23, 51]. EEG connectivity was calculated using the debiased estimator of the weighted phase lag index (wPLI). The wPLI is considered a conservative measure of phase synchronization that is robust against volume conduction, non-brain-related artefacts, and common reference artefacts [52]. This measure is also believed to have good test-retest reliability [53].

### 2.7 Statistical analyses

This study is a secondary analysis based on a patient cohort whose clinical outcomes have been reported elsewhere (manuscript under review at Communications Medicine). The present work focused on the analysis of EEG data from this cohort to establish relationships with clinical efficacy based on neural signatures. All EEG variables were confirmed to be not normally distributed. Accordingly, between-group differences (migraineurs *vs.* healthy controls) in PAF and functional connectivity were tested using the Mann-Whitney U test, and significance levels were adjusted using the false discovery rate (FDR) to address multiple comparisons. Finally, partial correlation analyses controlling for pain duration, pain-free days, gender, and medication status were performed to examine relationships between EEG indices and clinical outcomes. To verify the validity of the correlational results, a permutation test with 5000 iterations was applied, which provides a robust, assumption-free framework for assessing whether an observed correlation reflects a true relationship rather than random noise [54]. Statistical significance was set at *p* < 0.05.

### 2.8 Supplementary analyses

We examined the influence of a series of covariates on our findings, including age, gender, pain duration, pain-free days before EEG, and medication status. Multiple linear regression was conducted for PAF and phase synchronisation separately. As our sample included both episodic (n = 14) and chronic migraine (n = 15), we further examined our results in these two subsamples by comparing each group with healthy controls using the Mann-Whitney U test. The same false discovery rate approach was applied to address multiple comparisons. In addition, we present the treatment effects on clinical outcomes in this sample in the **Supplementary material**.

## 3. Results

### 3.1 Group difference in PAF

As shown in Fig. 2A, the normalized power spectra illustrate the distribution of power and the location of the PAF peak in a representative migraine patient and a healthy control participant, while clean EEG traces are presented in Fig. 2B. Migraineurs had a slower PAF in the right occipital cortex (O2) compared to healthy controls (9.81 ± 1.06 Hz *vs.* 10.46 ± 1.22 Hz; U = 288.50, *p*_FDR_ = 0.040, *Z* = −2.06) (Fig. 2C). In contrast, only a trend was observed in the left occipital cortex (O1, *p*_FDR_ = 0.088).

**Fig. 2.** PAF illustration and comparison. (A) Normalized power (μV^2^/Hz) as a function of frequency (Hz) for Migraine Patient A and Control Participant A, illustrating the distribution of power and the PAF within a frequency range of 0.05 to 15 Hz. (B) Averaged EEG trace from the O2 channel for Migraine Patient A and Control Participant A, showing amplitude (μV) over time (s); the trace highlights differences in the amplitude of neural oscillations between the two groups. (C) Group comparisons of PAF measured at occipital electrodes O1 and O2 using the Mann-Whitney U test. The figure presents PAF values for migraine patients compared to healthy participants. Error bars represent standard errors. PAF: peak alpha frequency; EEG: electroencephalography; O1: left occipital cortex; O2: right occipital cortex; ns: no significant difference. * indicates *p* < 0.05.

### 3.2 Group difference in phase synchronization

Compared to healthy controls, migraineurs showed significantly higher alpha-band phase synchronization between O2 and multiple frontal cortex electrode sites, including Frontal 1 (F1, U = 621, *Z* = −3.50, *p*_FDR_ = 0.025), Frontal 3 (F3, U = 608, *Z* = −3.84, *p*_FDR_ = 0.007), Frontal 4 (F4, U = 595, *Z* = −4.04, *p*_FDR_ = 0.003), Frontal 5 (F5, U = 639, *Z* = −3.36, *p*_FDR_ = 0.043), Frontal 6 (F6, U = 605, *Z* = −3.89, *p*_FDR_ = 0.006), Anterior Frontal 3 (AF3, U = 630, *Z* = −3.50, *p*_FDR_ = 0.025), Anterior Frontal 4 (AF4, U = 616, *Z *= −3.72, *p*_FDR_ = 0.011), and Frontal-Central 3 (FC3, U = 635, *Z* = −3.42, *p*_FDR_ = 0.034). A similar pattern of higher alpha-band synchronization was observed between O2 and parietal/occipital cortex sites, including Parietal 3 (P3, U = 621, *Z* = −3.64, *p*_FDR_ = 0.015), Parietal 5 (P5, U = 615, *Z* = −3.73, *p*_FDR_ = 0.010), Parieto-occipital 3 (PO3, U = 593, *Z* = −4.01, *p*_FDR_ = 0.003), and Parieto-occipital 7 (PO7, U = 615, *Z* = −3.55, *p*_FDR_ = 0.021) (Fig. 3). Migraineurs also showed significantly higher alpha-band phase synchronization between O1 and PO3 (U = 597, *Z* = −4.01, *p*_FDR_ = 0.003), as well as between O1 and Occipital Zero (Oz, U = 638, *Z* = −3.37, *p*_FDR_ = 0.040) (Fig. 3).

**Fig. 3.** Results of phase synchronization. Brain functional connectivity maps based on the O2 and O1 channels showing significant alterations in migraineurs. Following FDR correction, Mann-Whitney U tests showed that migraineurs demonstrated higher alpha-band phase synchronization between O2 and frontal regions (F1/F3/F4/F5/F6/AF3/AF4/FC3; *p*_FDR_ = 0.007–0.043), as well as between O2 and parietal regions (P3/P5/PO3/PO7; *p*_FDR_ = 0.003–0.021), relative to healthy controls. Migraineurs also showed significantly higher alpha-band phase synchronization between the left occipital cortex (O1) and parieto-occipital cortex (PO3)/occipital zero cortex (Oz) (*p*_FDR_ = 0.003–0.040). Connectivity strength is reflected by line thickness (0.0996–0.2198).

### 3.3 Correlation with treatment response

Permutation tests indicated that baseline phase synchronisation of parieto-occipital connections (O2_P3: *r* (23) = −0.34, *p* = 0.049, 95% Confidence Interval (CI) = [−0.698, 0.094]; O2_PO3: *r* (23) = −0.37, *p* = 0.036, 95% CI = [−0.706, 0.083]; O1_Oz: *r* (23) = −0.39, *p* = 0.026, 95% CI = [−0.760, −0.053]) was associated with the reduction rate in pain intensity at post-treatment, which was calculated as [(post-treatment pain intensity − baseline pain intensity)/baseline pain intensity] × 100% (Fig. 4). This relationship was identified when the two treatments were pooled together for migraineurs. Only migraineurs were included in the correlational analyses, as healthy controls did not receive treatment. No other significant relationships were identified between EEG indices and clinical outcomes.

**Fig. 4.** Correlation between neural activity and treatment outcomes. Partial correlations revealed relationships between baseline phase synchronization and the reduction in post-treatment pain intensity. All reported correlations were validated by a permutation test (5000 iterations). (A) O1_Oz (*r* = −0.39, *p* = 0.026). (B) O2_PO3 (*r* = −0.37, *p *= 0.036). (C) O2_P3 (*r *= −0.34, *p* = 0.049).

### 3.4 Supplementary results

In the covariate analyses for PAF, the regression model remained non-significant after controlling for the covariates (O1: *F*(5, 23) = 0.37, *p* = 0.863; O2: *F*(5, 23) = 0.44, *p* = 0.819), with adjusted *R*^2^ values of −0.13 for O1 and −0.11 for O2. Importantly, none of the covariates showed a significant independent association with PAF at either electrode (from O1 to O2, age: *p* = 0.466 and 0.701; pain duration: *p* = 0.823 and 0.539; pain-free days: *p* = 0.472 and 0.357; gender: *p* = 0.714 and 0.889; medication status: *p* = 0.850 and 0.610), indicating that these variables were not significant confounders for PAF.

In the covariate analyses of phase synchronisation, the regression model was also non-significant after adjustment (O1: *F*(5, 23) = 0.31, *p* = 0.905; O2: *F*(5, 23) = 0.11, *p* = 0.990), with adjusted *R*^2^ values of −0.14 for O1 and −0.19 for O2. Similarly, none of the covariates demonstrated a significant independent association with phase synchronisation at either electrode (from O1 to O2, age: *p* = 0.787 and 0.857; pain duration: *p* = 0.386 and 0.681; pain-free days: *p* = 0.805 and 0.801; gender: *p* = 0.868 and 0.676; medication status: *p* = 0.528 and 0.947), suggesting that these variables did not act as significant confounders for phase synchronisation.

In the episodic migraine subgroup analyses, the Mann-Whitney U test indicated that episodic migraine exhibited a significantly slower PAF than healthy controls at O2 (9.81 ± 1.06 Hz *vs.* 10.46 ± 1.22 Hz; U = 119.50, *p* = 0.031), whereas the difference at O1 did not reach significance (*p* = 0.097). Episodic migraine also showed significantly higher phase synchronisation between O2 and F3 (U = 491, *Z* = −3.80, *p* = 0.007), F4 (U = 500, *Z* = −3.56, *p* = 0.020), AF4 (U = 507, *Z* = −3.38, *p* = 0.039), Anterior Frontal 8 (AF8, U = 509, *Z* = −3.33, *p* = 0.047), FC3 (U = 507, *Z* = −3.38, *p* = 0.039), PO3 (U = 493, *Z* = −3.75, *p* = 0.010), P5 (U = 508, *Z* = −3.36, *p* = 0.043), and PO7 (U = 503, *Z* = −3.49, *p* = 0.027). In addition, higher phase synchronization was observed for O1, including its connections with PO3 (U = 500, *Z* = −3.56, *p* = 0.019) and Oz (U = 506, *Z* = −3.41, *p* = 0.035).

In the chronic migraine subgroup, no significant differences in PAF were observed at either O1 (*p* = 0.264) or O2 (*p* = 0.233). Similarly, no significant differences in phase synchronization were observed between chronic migraine and healthy controls.

## 4. Discussion

This study aimed to identify abnormal neural oscillations in migraineurs and evaluate their predictive value for rTMS treatment response. Our findings showed that migraineurs exhibited a slower eyes-open PAF than healthy controls. In addition, migraineurs demonstrated enhanced alpha-band functional connectivity both within parieto-occipital regions and between fronto-occipital areas. Baseline connectivity in the O2_P3, O2_PO3, and O1_Oz connections showed an uncorrected association with pain reduction at 1month post-treatment; however, most regions did not show an association with the analgesic response.

This study showed a slower PAF in migraineurs compared to healthy controls. Previous studies have identified a slower PAF when healthy participants were exposed to painful stimuli [17, 41, 55], and a larger body of work has further shown that lower PAF is associated with chronic pain conditions, including neuropathic pain and chronic low back pain [18, 30, 56, 57]. In migraine, slower PAF has also been reported to be associated with longer disease duration and longer headache attack duration [19]. Building on these, our data demonstrated a slower PAF in migraineurs relative to healthy controls.

In addition to PAF, migraineurs are characterised by alpha-band hyperconnectivity between parieto-occipital regions and between fronto-occipital areas. Although a wide range of abnormal oscillations has been reported in migraineurs, a large study of approximately 800 migraineurs identified occipital power at 12–13 Hz as a reliable oscillatory feature of migraine, and this feature ranked highest in the prediction model discriminating migraineurs from controls [23]. Our findings extend this evidence by demonstrating hyperconnectivity within the alpha frequency range. Previous studies indicated that migraine-associated vertigo is linked to a higher incidence of photic-driven EEG responses to stimulation in the alpha range [58, 59], and visually evoked EEG responses to 12 Hz stimulation have been found to increase before a migraine attack [60]. Together, these findings support cortical hyperexcitability to alpha-range stimulation and its potential relevance to migraine symptoms, including sensory sensitivity, photophobia, and pain. Moreover, our data adds spatial detail by identifying parieto-occipital and fronto-occipital patterns of alpha-band coherence [23]. Therefore, these findings suggest that, in migraine, the occipital cortex may be abnormally over-coupled with attentional and pain-related networks, which may contribute to cortical hyperexcitability and altered sensory processing in migraineurs.

The observation that these electrophysiological signatures, *i.e.*, slower PAF and alpha-band hyperconnectivity, were more pronounced in episodic migraineurs than in chronic patients may help to elucidate mechanisms underlying migraine progression. The enhanced alpha-band hyperconnectivity in episodic migraineurs is consistent with a state of pathological cortical hypersynchronization and reduced inhibitory control, which may promote abnormal spread of neural excitability across parieto-occipital and fronto-occipital networks, thereby contributing to the heightened sensory sensitivity and photophobia characteristic of the disorder [61, 62, 63]. In contrast, the attenuation of these signatures in chronic migraine may indicate disruption or maladaptive remodelling of large-scale oscillatory networks [64, 65, 66]. One possibility is that, as migraine becomes chronic, the brain shifts toward a more persistent and diffuse sensitization state, in which distinct, phasic pathological rhythms (such as alpha hypersynchrony) are less readily expressed and are replaced by a more generalised, less oscillation-specific pattern of dysregulation [67, 68, 69].

In a relatively small sample, we provided evidence for correlating neural connectivity with rTMS analgesic effects. Baseline hyperconnectivity between the parieto-occipital regions (O2_P3, O2_PO3, O1_Oz) was associated with a larger decrease in pain intensity at 1-month post-treatment. Using functional magnetic resonance imaging (fMRI), one previous migraine study found that DLPFC-rTMS increased functional connectivity between the frontal cortex and the somatosensory and cingulate cortices, which are involved in the processing of pain experiences [70]. In this way, our findings support the view that rTMS analgesic effects are associated with cortical hyperexcitability and altered sensory processing in migraineurs. In addition, although rTMS increased PAF in experimentally induced pain, it did not lead to a direct reduction in pain intensity [20]. Our data extend this observation by showing that, in migraineurs, parieto-occipital connectivity correlated with the analgesic effects of rTMS. However, it should be noted that two different rTMS protocols were included in this study, and these protocols differ in temporal profile and session scheduling.

These novel findings have implications for the management of migraine. rTMS treatment protocols need to be optimized to achieve better treatment efficacy, including the development of accelerated TMS paradigms [12, 71, 72, 73, 74]. Our data identified a predictive value of parieto-occipital connectivity for rTMS treatment in migraineurs. This finding therefore provides biomarkers that may inform the development of rTMS protocols and parameters to achieve improved analgesic efficacy. Moreover, connectivity-based TMS targeting has been suggested to be advantageous in the treatment of depression [75, 76, 77], and this concept has also been proposed for pain management in review studies [78, 79]. The current study identified parieto-occipital connectivity as a predictor of treatment effect, and this measure could be utilised to improve treatment efficacy for migraineurs. In addition, the occipital–frontal pathway observed in our data indicates the potential utility of dual-site TMS (ds-TMS). By targeting two brain regions, ds-TMS has been suggested to recruit more neural circuits or stronger inter-regional communication, which may be beneficial for patients [80, 81, 82].

It is worth noting that PAF is considered a reliable cortical biomarker for the study of pain [13, 14, 15], and it is unlikely to be substantially affected by EEG processing pipelines. However, variability in methodological choices can influence PAF estimates, including channel selection and calculation methods [13]. In this study, we selected occipital regions that have been shown to differentiate migraineurs from healthy controls [23]. Moreover, we used the peak-picking method for PAF calculation, which is more suitable for identifying individual differences [13], particularly in the context of group comparisons.

There were several limitations in this study. Resting EEG data were collected without consideration of migraine cycles, and there is evidence that different migraine phases may be associated with distinct neural oscillations [19, 22, 83]. Future studies may wish to validate our findings in the context of migraine cycles. EEG data were not collected following rTMS treatment, which limited our ability to investigate treatment-related changes in neural oscillations, and this is particularly relevant given that correlations with clinical outcomes were observed only at post-treatment. In addition, data from iTBS and 10-Hz rTMS were combined in the correlational analysis; although this was not ideal, it increased the sample size to some extent. Moreover, our clinical data indicated that these two protocols have comparable analgesic effects, and they are both excitatory in nature and share a mechanism involving increased gamma oscillation [13, 84]. However, we acknowledge that these protocols differ in temporal profile, session scheduling, and potentially neurophysiological effects. This study also focused on a limited set of channels that are critical to migraine, which may have concealed other regions as potential alternatives. Future studies could address these possibilities using data-driven approaches in larger samples. It should be noted that we did not observe group differences in alpha power, which may be related to our relatively small sample size compared with the earlier large study [23]. In addition, although a few studies have calculated PAF from eyes-open resting EEG data [56, 85], most studies in the literature have used eyes-closed resting EEG data [13], and we acknowledge this deviation from the prevailing approach. Resting EEG recorded for 5 minutes may also be relatively short, and longer recordings could be considered to increase the signal-to-noise ratio. Finally, although permutation testing indicated a significant correlation between phase synchronisation and pain intensity, we acknowledge that the sample size may be underpowered for reliable prediction.

## 5. Conclusions

This study identified distinct neural oscillation abnormalities in migraineurs compared with healthy controls. Migraineurs exhibited a significantly slower PAF in the right occipital cortex and demonstrated widespread alpha-band hyperconnectivity, particularly between parieto-occipital and fronto-occipital regions. Importantly, baseline hyperconnectivity in specific parieto-occipital connections (O2_P3, O2_PO3, and O1_Oz) predicted greater reductions in pain intensity after excitatory rTMS targeting the left DLPFC. These findings indicate that PAF slowing and parieto-occipital alpha hyperconnectivity may serve as oscillatory biomarkers in migraine. More importantly, the predictive value of parieto-occipital connectivity for rTMS analgesic efficacy highlights its potential utility for optimising rTMS treatment protocols and personalising therapeutic strategies for migraine management.

## Supplementary Material

Supplementary material associated with this article can be found, in the online version, at https://files.jofph.com/ files/article/2054082147923181568/attachment/ Supplementary%20material.docx.


