# Supplementary material for: Identifying neural oscillation and phase synchronization abnormalities in migraine and their predictive values in transcranial magnetic stimulation
Source: J Oral Facial Pain Headache. 2026 May 12;40(3):118–29. doi: 10.22514/jofph.2026.041 (PMC13223903; doi:10.22514/jofph.2026.041)
Supplement: Supplementary file 1 [file Supplementary-material.docx]

Supplementary material

# Supplementary Results S1: covariate analyses of Peak Alpha Frequency (PAF) and phase synchronization

We examined the influence of a series of covariates on our findings, including age, gender, pain duration, pain-free days before EEG, and medication status. Specifically, multiple linear regression was conducted on PAF and phase synchronization separately.

## S1.1 Multiple linear regression on PAF

In covariate analyses for PAF, the regression model was not significant (left occipital cortex (O1): *F*(5, 23) = 0.37, *p* = 0.863; right occipital cortex (O2): *F*(5, 23) = 0.44, *p* = 0.819) after controlling for the covariates, with an adjusted *R*² of −0.13 for O1 and −0.11 for O2. Critically, none of the covariates individually demonstrated a significant effect on the outcome measure (from O1 to O2, age: *p* = 0.466 and 0.701; pain duration: *p* = 0.823 and 0.539; pain-free days: *p* = 0.472 and 0.357; gender: *p* = 0.714 and 0.889; medication status: *p* = 0.850 and 0.610.

## S1.2 Multiple linear regression on phase synchronization

In the analyses of phase synchronization, the regression model was not significant (O1: *F*(5, 23) = 0.31, *p* = 0.905; O2: *F*(5, 23) = 0.11, *p* = 0.990) after controlling for the covariates, with an adjusted *R*² of −0.14 for O1 and −0.19 for O2. None of the covariates individually demonstrated a significant effect on the outcome measure (from O1 to O2, age: *p* = 0.787 and 0.857; pain duration: *p* = 0.386 and 0.681; pain-free days: *p* = 0.805 and 0.801; gender: *p* = 0.868 and 0.676; medication status: *p* = 0.528 and 0.947). These results indicate that these variables did not function as significant confounders for phase synchronization.

# Supplementary Information Section S2: Subgroup Analysis of Peak Alpha Frequency (PAF) and phase synchronization

As our sample included both episodic (n = 14) and chronic migraine (n = 15), we further examined our results in these two subsamples, by comparing them with healthy controls with Mann-Whitney U test. The same false discovery rate was used to address multiple comparisons.

## S2.1 Subgroup analysis: episodic migraineurs

In the episodic migraine analyses, Mann-Whitney U test indicated that episodic migraine had a significantly slower PAF compared to healthy controls in O2 (9.81 ± 1.06 Hz *vs.* 10.46 ± 1.22 Hz; U = 119.50, *p* = 0.031) but not in the O1 (*p* = 0.097). Similarly, episodic migraine showed significantly higher phase synchronization between O2 and Frontal 3 (F3, U = 491, *Z* = −3.80, *p* = 0.007), Frontal 4 (F4, U = 500, *Z* = −3.56, *p* = 0.020), Anterior Frontal 4 (AF4, U = 507, *Z* = −3.38, *p* = 0.039), Anterior Frontal 8 (AF8, U = 509, *Z* = −3.33, *p* = 0.047), and Frontal-Central 3 (FC3, U = 507, *Z* = −3.38, *p* = 0.039), Parieto-occipital 3 (PO3, U = 493, *Z* = −3.75, *p* = 0.010), Parietal 5 (P5, U = 508, *Z* = −3.36, *p* = 0.043), and Parieto-occipital 7 (PO7, U = 503, *Z* = −3.49, *p* = 0.027). Furthermore, a significant higher phase synchronization was also identified for O1, with PO3 (U = 500, *Z* = −3.56, *p* = 0.019) and Occipital Zero (Oz, U = 506, *Z* = −3.41, *p* = 0.035).

## S2.2 Subgroup analysis: chronic migraineurs

In chronic migraine, no significant differences in PAF were observed for either the O1 (*p* = 0.264) or O2 (*p* = 0.233). Similarly, no significant differences in phase synchronization were observed between chronic migraine and healthy controls.

# Supplementary Information Section S3: Linear Mixed Mode Analysis of Clinical Outcomes

Clinical outcomes of this cohort have been reported elsewhere (manuscript under review at Communications Medicine). We thus analysed the clinical outcomes in this sample (n = 29, 16 in 10 Hz and 13 in intermittent theta burst stimulation (iTBS)) to demonstrate the treatment effects. We analysed the data using linear mixed-effects models (LMM), which are more robust to violations and provide a flexible framework for modelling complex data structures. Fixed effects included treatment, time point, and the interaction effect. Outcomes at each visit were considered as the dependent variable. Comparisons were conducted using a Bonferroni correction at *p* < 0.05.

LMM analysis revealed a significant time effect on headache frequency (*F*(2, 54) = 19.93, *p* = 0.001), in which two treatments were equally effective in reducing headache days from Pre- to Post-treatment (Mean difference = −6.13, *p* = 0.001, 95% Confidence Interval (CI) = [2.24, 10.01]) and maintained this effect to Follow-up (Mean difference = −9.78, *p* = 0.001, 95% CI = [5.89, 13.67]). However, there was no significant treatment × time interaction effect on headache frequency (*F*(2, 54) = 0.21, *p* = 0.815) (**Supplementary Fig. 1A**).

In terms of pain intensity, LMM analysis also revealed a significant time effect (*F*(2, 54) = 30.11, *p* = 0.001), whereby two treatments were equally effective in reducing pain intensity from Pre- to Post-treatment (Mean difference = −2.88, *p* = 0.001, 95% CI = [1.82, 3.94]) and maintained this effect to Follow-up (Mean difference = −2.86, *p* = 0.001, 95% CI = [1.81, 3.92]). The interaction effect was not significant (*F*(2, 54) = 2.89, *p* = 0.064) (**Supplementary Fig. 1B**).

LMM revealed a significant time effect on short-form McGill Pain Questionnaire (SF-MPQ) (*F*(2, 54) = 18.90, *p* = 0.001). *Post-hoc* tests indicated that two treatments were equally effective in reducing SF-MPQ from Pre- to Post-treatment (Mean difference = −6.80, *p* < 0.001, 95% CI = [3.29, 0.57]). This effect was maintained to Follow-up (Mean difference = −8.15, *p* < 0.001, 95% CI = [3.29, 10.31]) (**Supplementary Fig. 1C**).

LMM analysis revealed a significant treatment × time interaction effect on Pittsburgh Sleep Quality Index (PSQI) (*F*(2, 54) = 3.38, *p* = 0.042). *Post-hoc* tests indicated that iTBS treatment improved sleep quality (Mean difference = −3.46, *p* = 0.027, 95% CI = [−6.62, −0.30]) from Pre- to Post-treatment. This effect was maintained to Follow-up (Mean difference = −5.46, *p* = 0.001, 95% CI = [−8.62, −2.30]). No such effect was observed for 10-Hz treatment (**Supplementary Fig. 1D**).

LMM analysis revealed a significant group effect of treatment on 7-item Generalized Anxiety Disorder scale (GAD) scores (*F*(2, 54) = 4.79, *p* = 0.037). *Post-hoc* tests indicated that the iTBS group showed significantly lower GAD scores overall compared to the 10-Hz group (Mean difference = −2.05, *p* = 0.037, 95% CI [−3.97, −0.13]). Additionally, a significant main effect of time was observed (*F*(2, 54)= 8.77, *p* = 0.001). *Post-hoc* tests demonstrated that GAD scores at both post-treatment (mean difference = −3.08, *p* = 0.008, 95% CI [−5.48, −0.68]) and Follow-up (mean difference = −3.85, *p* = 0.001, 95% CI [−6.25, −1.44]) were significantly reduced compared to Pre-treatment (**Supplementary Fig. 1E**).

In 9-item Patient Health Questionnaire (PHQ), LMM analysis revealed a significant time effect (*F*(2, 54) = 16.53, *p* = 0.001). *Post-hoc* tests indicated that two treatments were equally effective in reducing depression from Pre- to Post-treatment (Mean difference = −3.71, *p* < 0.001, 95% CI = [−5.96, −1.46]). This effect was maintained to Follow-up (Mean difference = −5.05, *p* < 0.001, 95% CI [−7.30, −2.80]) (**Supplementary Fig. 1F**).


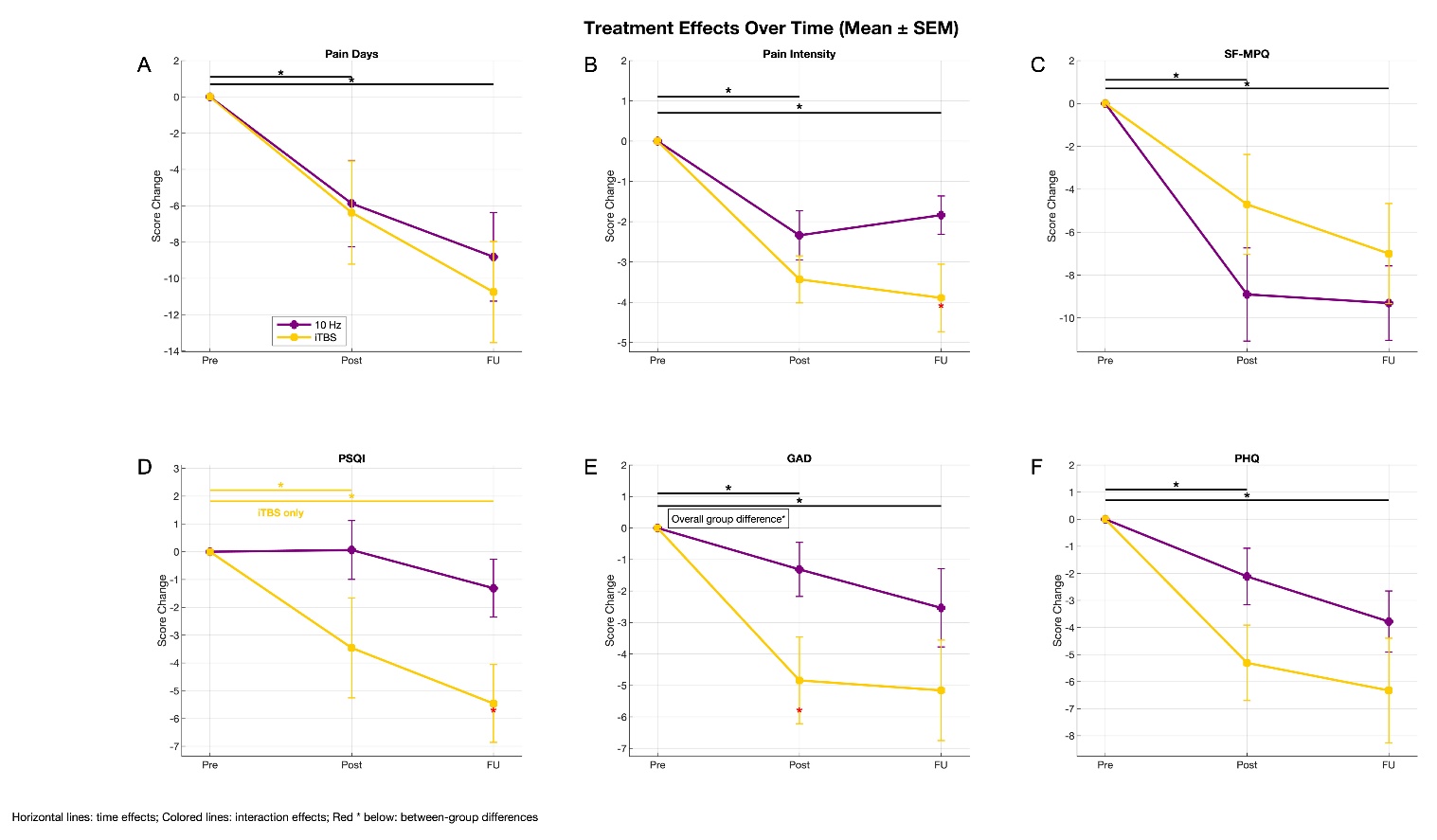


**Supplementary Fig. 1.** **Treatment effects on outcome measures.** Pre indicates pre-treatment, Post indicates post-treatment, FU indicates follow-up. (A) a significant time effect on headache frequency. (B) a significant time effect on pain intensity. (C) a significant time effect on short-form McGill Pain Questionnaire (SF-MPQ). (D) a significant treatment × time interaction effect on Pittsburgh Sleep Quality Index (PSQI). (E) a significant group effect of treatment on 7-item Generalized Anxiety Disorder scale (GAD). (F) a significant time effect on 9-item Patient Health Questionnaire (PHQ). SEM: Standard Error of the Mean; iTBS: intermittent theta burst stimulation; FU: follow-up Post. *****: indicates *p* < 0.05.
